# Inhibitory effects and molecular mechanisms of tetrahydrocurcumin against human breast cancer MCF-7 cells

**DOI:** 10.3402/fnr.v60.30616

**Published:** 2016-02-17

**Authors:** Xiao Han, Shan Deng, Ning Wang, Yafei Liu, Xingbin Yang

**Affiliations:** Key Laboratory of Ministry of Education for Medicinal Resource and Natural Pharmaceutical Chemistry, College of Food Engineering and Nutritional Science, Shaanxi Normal University, Xi'an, China

**Keywords:** tetrahydrocurcumin, antitumor effect, cell cycle, apoptosis, mitochondria

## Abstract

**Background:**

Tetrahydrocurcumin (THC), an active metabolite of curcumin, has been reported to have similar biological effects to curcumin, but the mechanism of the antitumor activity of THC is still unclear.

**Methods:**

The present study was to investigate the antitumor effects and mechanism of THC in human breast cancer MCF-7 cells using the methods of MTT assay, LDH assay, flow cytometry analysis, and western blot assay.

**Results:**

THC was found to have markedly cytotoxic effect and antiproliferative activity against MCF-7 cells in a dose-dependent manner with the IC_50_ for 24 h of 107.8 μM. Flow cytometry analysis revealed that THC mediated the cell-cycle arrest at G0/G1 phase, and 32.8% of MCF-7 cells entered the early phase of apoptosis at 100 μM for 24 h. THC also dose-dependently led to apoptosis in MCF-7 cells via the mitochondrial pathway, as evidenced by the activation of caspase-3 and caspase-9, the elevation of intracellular ROS, a decrease in Bcl-2 and PARP expression, and an increase in Bax expression. Meanwhile, cytochrome C was released to cytosol and the loss of mitochondria membrane potential (Δψm) was observed after THC treatment.

**Conclusion:**

THC is an excellent source of chemopreventive agents in the treatment of breast cancer and has excellent potential to be explored as antitumor precursor compound.

Breast cancer is the most common cancer in women, and it is the second leading cause of death in women after lung cancer (1). Large-scale epidemiological cohort studies have shown that dietary patterns, foods, and nutrients are closely associated with the risk for breast cancer (2). Indeed, studies have also shown that the ingestion of fruits and vegetables may reduce the risk of cancer, cardiovascular disease, and immunodysfunctions (2, 3). Due to the advantage of low toxicity and high efficacy, plant-derived foods play an increasing role in cancer chemoprevention and therapy (4). In recent years, naturally occurring 1,7-bis(4-hydroxy-3-methoxyphenyl)-1E,6E-heptadiene-3,5-dione (curcumin) has been strongly investigated as the promising anticancer agent (5–8). Curcumin is a natural flavonoid and has been derived from the *Curcuma longa*, which is commonly used as a food-coloring agent (5). Interestingly, numerous studies suggested that curcumin had significant antitumor activities *in vitro*, while the antitumor effects *in vivo* were not significant, which might be related to its poor absorption (9, 10).

Tetrahydrocurcumin (THC), a major metabolite of curcumin, is a natural polyphenol in *C. longa*, and it has the identical β-diketone structure and phenolic groups, but curcumin lacks the double bonds (Fig. 1a) (11). Unlike curcumin, THC is stable in phosphate buffer saline at various pH values, and it is easily absorbed by the gastrointestinal tract, suggesting that THC may also play a crucial role in curcumin-induced biological effects, and may have the excellent potential to be developed into an antitumor precursor compound (12). However, the anticancer effects of THC remain poorly understood.

**Fig. 1:**
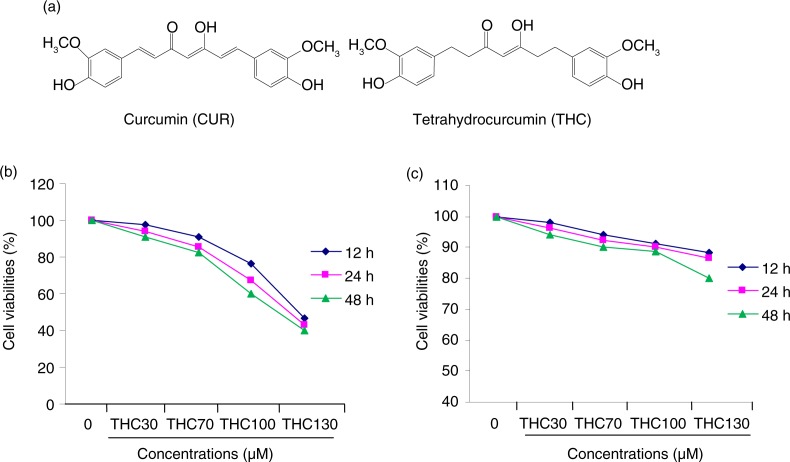
Inhibitory effects of THC on cell proliferation in human breast carcinoma MCF-7 cells. (a) Chemical structures of THC and its parent curcumin (CUR), (b) antiproliferative effects of THC against MCF-7 cells, and (c) normal mammary epithelial cell H184B5F5/M10 cells. MCF-7 cells were treated with or without different concentrations of THC (0, 30, 70, 100, and 130 µM) for 12, 24, and 48 h, respectively, and then the cell viability was assessed by MTT assay. Results are mean values±SD of three independent experiments. *p<0.05 and **p<0.01 indicate statistically significant difference versus control group.

The present study was therefore designed to demonstrate the cytotoxicity of THC derived from *C. longa* in human breast cancer MCF-7 cell line, and to further investigate the possible cellular mechanisms by evaluating cell cycle distribution, intracellular ROS generation, and mitochondrial dysfunction. Our results suggested that THC induced G0/G1 arrest and apoptosis via the mitochondrial pathway in MCF-7 cells. These findings have important implications for the potential use of the promising THC as a therapeutic or prophylactic treatment for cancer in humans.

## Materials and methods

### Materials and chemicals

Curcumin (CUR) and THC were obtained from Wuhan reagent company (Wuhan, China). Dimethylsulfoxide (DMSO), 5-fluoro-2,4 (1 h,3 h) pyrimidinedione (5-FU), 3-(4,5-dimethylthiazol-2-yl)-2,5-diphenyltetrazolium bromide (MTT), rhodamine 123 (Rh123), ethylenediaminetetraacetic acid (EDTA), RNAse-A and propidium iodide (PI), phenylmethyl-sulfonyl fluoride (PMSF), 4′,6-diamidino-2-phenylindole (DAPI), electro-chemi-luminescence (ECL), dichlorofluorescein diacetate (DCFH-DA), and tris-HCl and glycine were obtained from Sigma-Aldrich (St, Louis, MO, USA). Assay kits of BCA, caspase-3, caspase-9, and lactate dehydrogenase (LDH) were purchased from Nanjing Jiancheng Bioengineering Institute (Nanjing, China). Phosphate buffer solution (PBS), 0.1% tween-20 in phosphate buffer solution (PBST), and sodium dodecyl sulfonate (SDS) were purchased from Zhengjiang Wanbang Pharmaceutical Co. (Wenling, China). The primary antibodies against Bax, Bcl-2, poly (ADP-ribose) polymerase (PARP), cytochrome c, and the horseradish peroxidase (HPR)-conjugated goat anti-mouse secondary antibody were provided by BioVision, Inc., (BioVisio, Palo Alto, CA, USA). All the other cell culture reagents were purchased from Sinopharm (Beijing, China), and all the other chemicals were of analytical grade.

### Cell line and cell culture

Human breast carcinoma cell MCF-7 line and normal mammary epithelial cell H184B5F5/M10 cell line were obtained from Cell Bank of Institute of Biochemistry and Cell Biology, Chinese Academy of Sciences (Shanghai, China). The cells were grown in RPMI 1640 medium with 10% fetal bovine serum and 1% immune body. The cells were incubated at 37°C in a humidified incubator containing with 5% CO_2_.

### Assay for cell viability

The effect of THC on cell viability was determined by the MTT assay as previously described with some modifications (13). Cells were grown in a 96-well plate for 24 h, and then the cells were incubated at 12, 24, and 48 h with various concentrations of THC. After the exposure period, 10 mL of MTT (5.0 mg/mL) in PBS solution was added to each well at a final concentration of 0.5 mg/mL and then the plate was further incubated for 4 h. The supernatants were aspirated carefully, and the MTT-formazan crystals formed by metabolically viable cells were dissolved in 150 µL of SDS. The absorbance was measured at 570 nm using an enzyme-linked immunosorbent assay (ELISA) reader (Rayto-RT6000, Guangdong, China).

### LDH assay for cytotoxicity

Cytotoxicity was evaluated by LDH after the treatment with THC. The leakage into the media of LDH, an indicator of cell membrane injury, was detected with an LDH kit (Jiancheng BioEngineering, Nanjing, China) according to the procedures described previously (14). Briefly, at the end of the incubation with indicated concentrations of THC for 24 h, 20 µL of culture supernatants of MCF-7 cells or H184B5F5/M10 cells were taken out for the assay of extracellular LDH, which could catalyze the conversion of lactate to pyruvate, and then reacted with 2, 4-dinitrophenylhydrazine to show the brownish red color in basic solution. After reaction, the absorbance of each sample was read at wavelength 450 nm, and the extracellular LDH activity was calculated as follows: LDH activity (U/L)=(OD_test_−OD_control_)/(OD_standard_−OD_blank_)×C_standard_×N_dilute times_×1,000.

### Morphological observation of nuclear change

The MCF-7 cells were treated with various concentrations of THC, and the treated cells were washed with PBS and fixed in 4% paraformaldehyde for 30 min at room temperature, and then washed with PBS. The fixed cells were incubated with 1.0 µg/mL DAPI solution for 10 min at room temperature in the dark. Stained solution was washed out with PBS, and then the cells were observed with a fluorescence microscope (Leica DMIL LED, Leica, Germany) for determination of nuclear morphological change (15).

### Cell cycle analysis

To determine the cell cycle distribution, MCF-7 cells were plated at a density of 1×10^5^ cells/well in six-well plate, and were grown for 24 h at 37°C and 5% CO_2_ in the presence of 0, 70 and 100 µM THC or 5-FU (50 µM). The cells were harvested and washed with PBS, and then the cells were fixed in 100 µL of iced 70% ethanol at −20°C. The cells were left to stand overnight and then collected by centrifugation. Afterwards, the cells were resuspended in 1.0 mL PBS and 50 µL ribonuclease and incubated at room temperature for 30 min, and then 50 µL PI was added and the cells were left to stand for another 30 min. The cell cycle distribution was measured with flow cytometry (Millopore corporation, Billerica, MA, USA) (16).

### Cell apoptosis assay

The extent of apoptosis was measured through Annexin V-FITC/PI double staining assay (17). Briefly, MCF-7 cells were treated with THC (0, 70, 100 µM) or 5-FU (50 µM) for 24 h, and then cells were collected, washed twice with PBS buffer, and stained with Annexin V-FITC and PI, followed by an assay using flow cytometry (Millopore corporation, Billerica, MA, USA). The number of intact cells, early apoptotic cells, and late apoptotic/necrotic cells were discriminated by counting the cells directly.

### ROS assay in MCF-7 cells

Intracellular ROS production as hydrogen peroxide (H_2_O_2_) was measured using dichlorofluorescein diacetate probes (18). Briefly, MCF-7 cells were seeded in 12-well plates and then exposed to a series concentration of THC for 24 h. After incubation, the cells were detached with trypsin-EDTA and washed twice with PBS. The cells were immediately resuspended in 0.2 mL PBS containing DCFH-DA at the final concentration of 10 µM, and reacted at 37°C for 30 min. ROS production of MCF-7 cells was evaluated by flow cytometry.

### Detection of mitochondrial membrane potential (Δψm)

The mitochondrial membrane potential (Δψm) was measured by flow cytometer using the cationic lipophilic green fluorochrome rhodamine 123 (19). MCF-7 cells were treated with various concentrations of THC for 24 h and the treated cells were harvested, washed twice with PBS, incubated with 1 mM rhodamine 123 at 37°C for 30 min, and washed twice with PBS. Fluorescence was determined by a Guava EasyCyte Plus Flow Cytometry System.

### Determination of caspase-3 and caspase-9 activities

Caspase-3 and caspase-9 activities were assessed by Caspase Assay Kit (Jiancheng BioEngineering, Nanjing, China) according to the manufacturer's instruction. Control or treated cells were lysed in 100 µL of cold lysis buffer containing 1.0 M dithiothreitol (DTT 10 µL/mL buffer) and incubated in ice for 15 min. After centrifugation, supernatant containing about 20–40 µg protein was mixed with 90 µL detection buffer and 10 µL catalytic substrate (ACDEVD-PNA specific for caspase-3 and AC-LEHD-PNA for caspase-9) in a 96-well microplate (20). All samples were incubated in 37°C for 2 h. The absorbance representing the activities of caspases was measured at 405 nm with ELISA reader (Rayto-RT6000, Guang-dong, China).

### Total protein extraction and western blot assays

MCF-7 cells were treated with THC at 70 and 100 µM for 24 h. For isolation of total protein fractions, the cells were collected, washed twice with cold PBS, and lysed with 0.1 mL of cold lysis buffer (150 mM NaCl, 50 mM of pH 7.4 Tris, 1 mM EDTA, 1% Trition X-100, 0.5% SDS, 0.01% PMSF) (21). The lysate was centrifuged at 12,000 g for 30 min at 4°C. Protein concentrations were determined with the BCA protein assay kit (Jiancheng BioEngineering, Nanjing, China). Total protein samples were separated by 12% SDS-PAGE and were transferred to a PVDF membrane. After non-specific binding sites were blocked with 5% non-fat dry milk in PBS for 60 min, transferred membrane was probed at 4°C overnight with primary antibodies (1:500). The membrane was washed with 0.1% tween-20 in PBS buffer, and then the membranes were probed for 1 h at room temperature with secondary antibody (1:5,000). Protein of interest was incubated with ECL detection reagent and developed by exposure to X-ray films. The protein expression was quantified densitometrically using Image J Software (version 1.43, National Institutes of Health, Bethesda, USA).

### Statistical analysis

All the present data are expressed as mean±SD of three independent experiments. The significant difference from the respective control for each experimental test was assessed by one-way analysis of variance (ANOVA) using SPSS 19.0 software. The difference is considered significant if *p*<0.05.

## Results

### THC inhibited cell proliferation of MCF-7 cells

The inhibitory effect of THC against human breast cancer MCF-7 cells was determined by MTT assay. The inhibitory rate of MCF-7 cells treated with THC at 30, 70, 100, and 130 µM for 12, 24, and 48 h was shown in Fig. 1b. It was found that THC significantly inhibited the growth of MCF-7 cells in a dose-dependent and time-dependent manner. However, the antiproliferative effect of THC on H184B5F5/M10 normal mammary epithelial cells was not observed at the same test concentration for 12, 24 and 48 h (Fig. 1c. The results of MTT assay showed that THC had a markedly antiproliferative activity against the MCF-7 cells in a dose-dependent manner with the IC_50_ for 24 h of 107.8 µM.

**Fig. 2:**
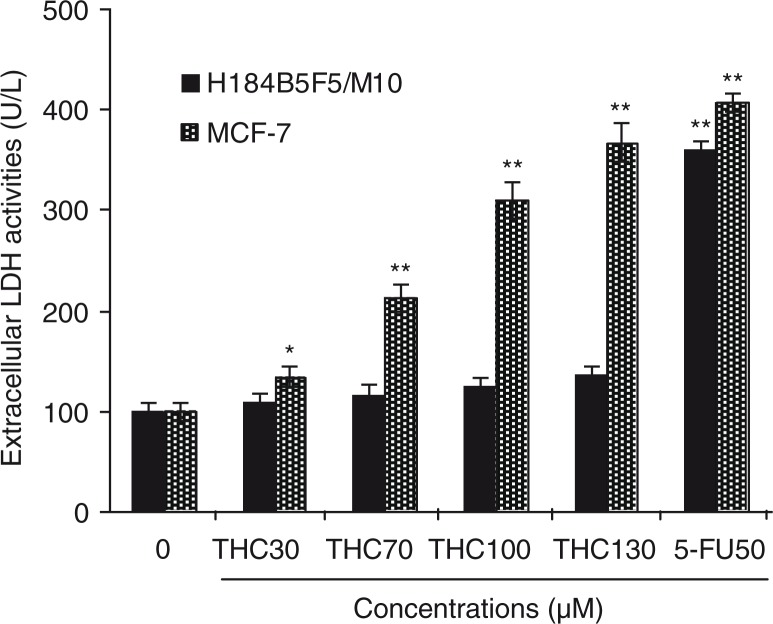
Cytotoxicity of THC to MCF-7 cells. MCF-7 cells were incubated with different concentrations of THC (0, 30, 70, 100, and 130 µM), and authorized 5-fluoro-2, 4 (1 h, 3 h) pyrimidinedione (5-FU, 50 µM) was used as positive control for 24 h, respectively. Cytotoxic effect was measured by LDH assay and expressed as U/L of LDH released from the MCF-7 cells after 24 h of incubation. All data were reported as the mean±SD of three separate experiments, and asterisks denote a statistical significance in comparison.

To further confirm the proliferation inhibitory effects of THC on MCF-7 cells, LDH assay was also performed as another indicator of THC-caused cytotoxicity to MCF-7 and H184B5F5/M10. As shown in Fig. 2, THC at 30, 70, 100, and 130 µM resulted in 1.3-, 2.1-, 3.1-, and 3.9-fold (*p*<0.01) increases over the untreated control in the LDH leakage from MCF-7 cells after 24 h of treatment, respectively. Moreover, compared with the untreated MCF-7 cells, the LDH leakage of 5-FU-treated MCF-7 cells has significantly increased, and the increase of THC at 100 and 130 µM is close to the increase level of 5-FU. However, this cytotoxic effect was not observed in normal H184B5F5/M10 cells. Obviously, the observations implied that MCF-7 cells exhibited a higher sensitivity to THC than H184B5F5/M10 cells, which were consistent with the MTT results. As a result, THC exerted high sensitivity to MCF-7 cancer cells and might be amenable to further tests for molecular mechanism of its anticancer effects.

### THC-induced cell cycle arrest of MCF-7 cells

**Fig. 3:**
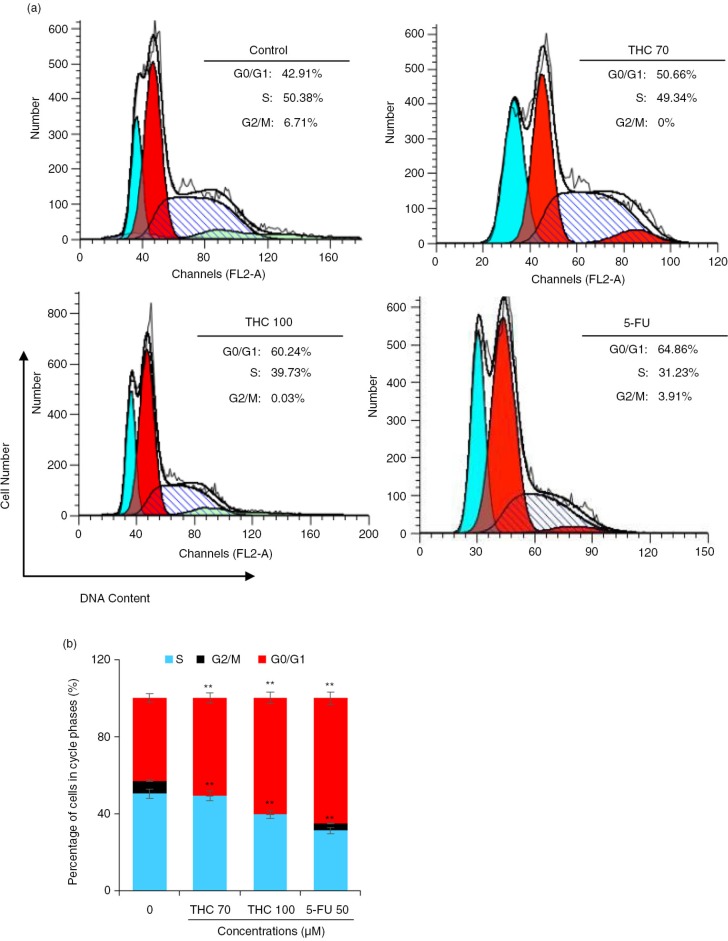
Effects of THC on MCF-7 cell cycle distribution. (a) MCF-7 cells were treated with the tested THC, and the collected cells were stained with propidium iodide (PI) for 24 h after fixation by 75% of ethanol, and analyzed for DNA content by flow cytometry. (b) Representative histograms of DNA content and cell cycle phases (G0/G1, S, and G2/M) in MCF-7 cells treated with 70, 100 µM THC and authorized 5-FU (50 µM) was used as positive control for 24 h, as shown. Horizontal and vertical axes indicate the relative nuclear DNA content and number of cells, respectively. Ratios of various cell cycle phases including G0/G1, S, and G2/M were presented. Data were shown as the means mean±SD, *p<0.05 and **p<0.01 as compared to control.

To determine the mechanism of the antiproliferative effect of THC, cell cycle progression in MCF-7 cells was investigated. The percentages of cell cycle were measured by flow cytometry (22). As shown in Fig. 3a, THC induced a strong G0/G1 phase arrest in a dose-dependent manner (*p*<0.05). When MCF-7 cells were incubated with 70 and 100 µM THC and 5-FU for 24 h, an accumulation of cell population in a G0/G1 phase increased from 42.9% of the control group to 50.7, 60.2 and 64.9%, accompanied by a decrease of the percentage of cells in the S phase from 50.4% of the untreated cells to 49.3, 39.7, and 31.2%, respectively. Interestingly, the results of accumulation of cell population in G0/G1 phase and S phase, which was treated with 100 µM THC is similar to treated with 5-FU. However, THC at the tested concentrations did not have significant impact on the percentage of G2/M phase cells (*p*<0.05, Fig. 3b). These results indicate that THC could result in G0/G1 phase arrest of MCF-7 cells, eventually inhibiting the growth of MCF-7 cells.

### Apoptotic induction on MCF-7 cells by THC

**Fig. 4:**
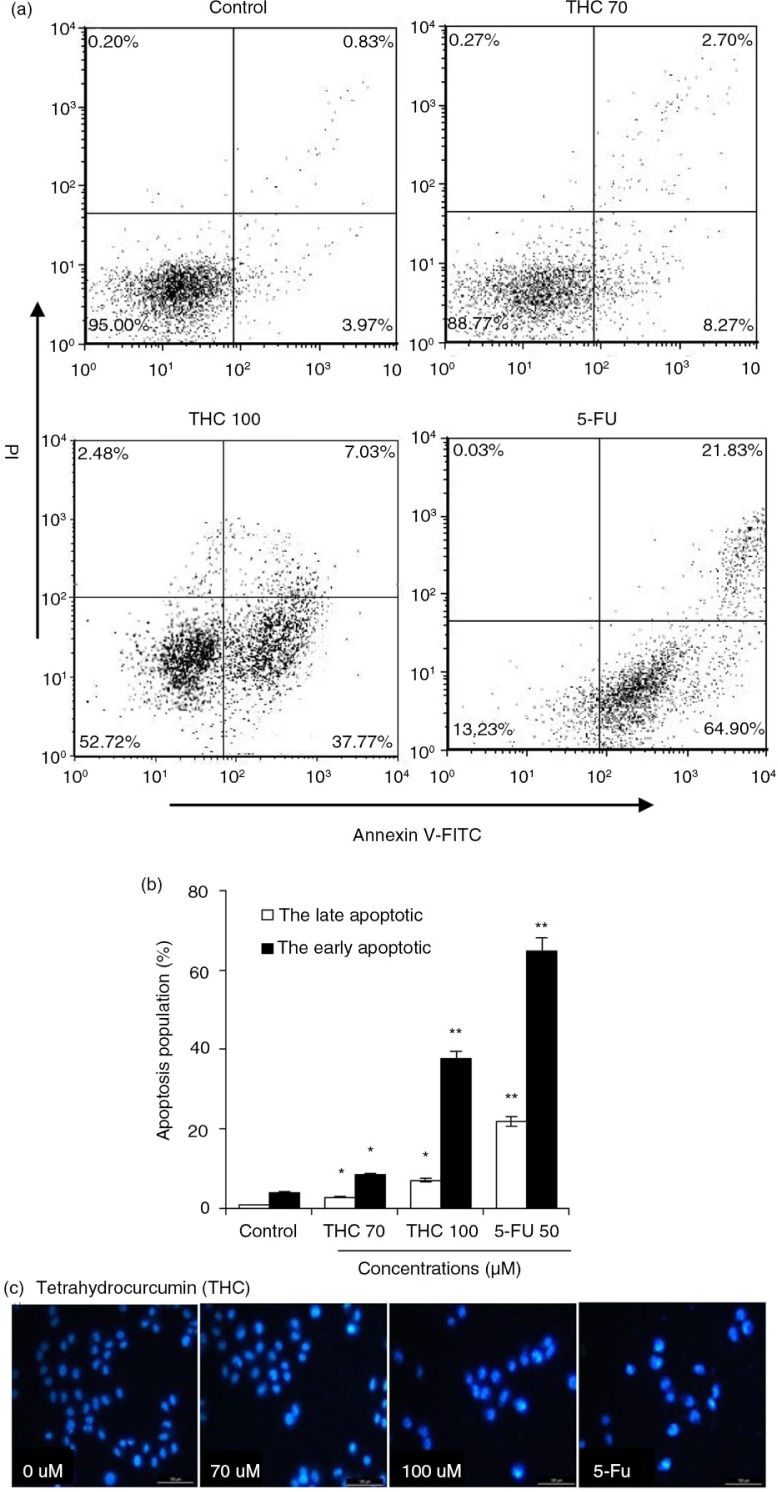
THC-induced apoptosis on MCF-7 cells. (a) MCF-7 cells were treated with different concentrations of THC (0, 70, and 100 µM), and authorized 5-FU (50 µM) was used as positive control. After 24 h of treatment, effects of THC on cell apoptosis necrosis of MCF-7 cells were assessed by flow cytometry. Representative dot plots of Annexin V-FITC/PI staining are shown for the tested compounds. (b) The percentage of apoptotic cells was calculated from the ratio of early apoptotic cells at 24 h of exposure of THC. (c) MCF-7 cells were treated with 0, 70, 100 µM THC for 24 h. Morphological changes were determined by fluorescence microscope. All experiments were done independently in triplicate per experimental point, and representative results are shown. Statistically significant difference is indicated at *p<0.05 or **p<0.01 level.

To further confirm the apoptotic induction ability of THC, MCF-7 cells were stained with Annexin V-FITC and PI, followed by flow cytometry analysis. As shown in Fig. 4a, the control cells showed normal cell viability without significant cell apoptosis. When MCF-7 cells were treated with 70, 100 µM THC or 5-FU, the percentage of early apoptotic cells significantly raised by 8.3, 37.8, and 64.9%, respectively, in contrast with control cells (*p*<0.05, *p*<0.01, Fig. 4b). As shown in Fig. 4b, THC at 70, 100 µM and 5-FU also caused 2.7, 7.0, and 21.8% of late necrotic/apoptotic cells, respectively. Furthermore, a DNA-binding dye, DAPI, was also used for nuclear staining to elucidate apoptotic cell death due to the exposure of MCF-7 cells to THC and 5-FU (Fig. 4c). Under a fluorescence microscope, the normal cells with nuclear staining exhibited intact and round-shape nuclei, characterized by the slight staining because of euchromatin in MCF-7 cells. In contrast, the exposure to THC at 70 and 100 µM for 24 h altered MCF-7 cellular nuclear morphology with increased, condensed, or fragmental chromatin, which is characteristic of apoptotic cells culminating in chromatin condensation and nuclear fragmentation (23). Besides, the cellular nuclear morphology with the treatment of 100 µM THC is close to the treatment of 5-Fu.

### THC triggered a marked loss of Δψm

**Fig. 5:**
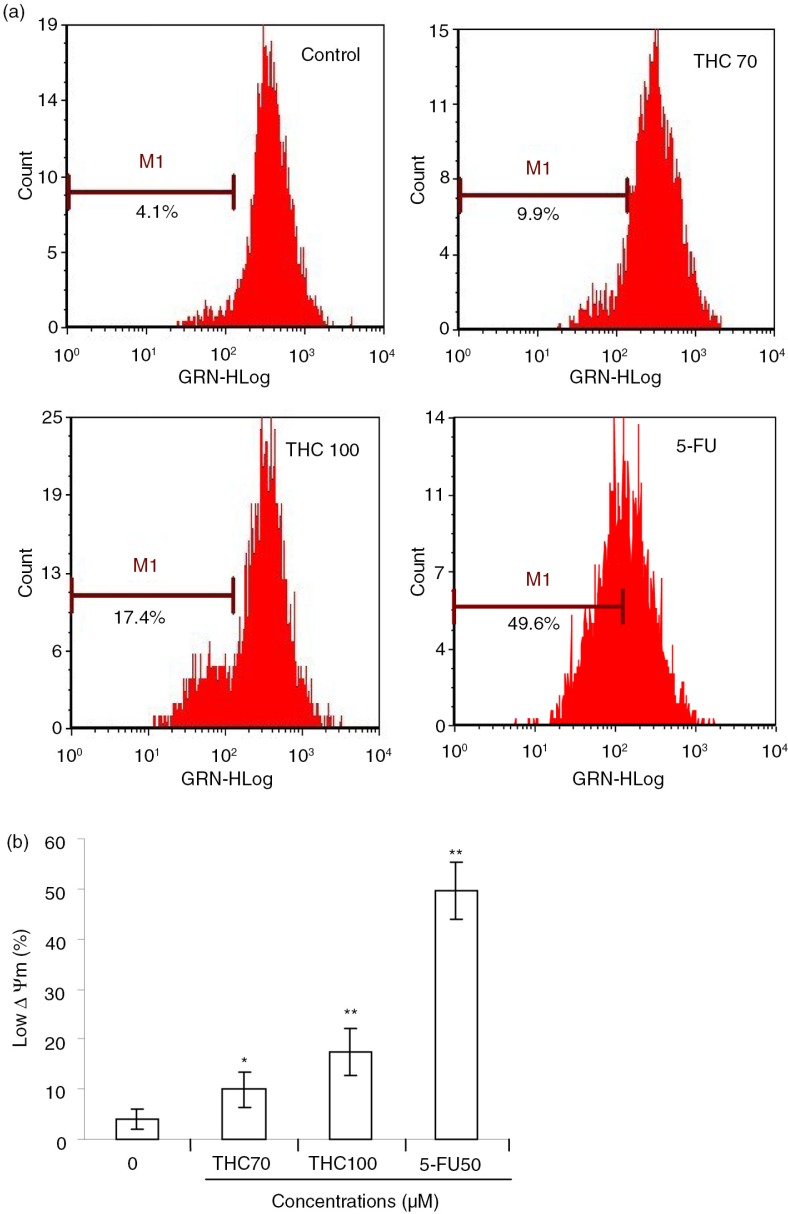
Effects of THC on mitochondrial membrane potential (Δψm) in MCF-7 cells. (a) Evaluation of mitochondria membrane potential (Δψm) in MCF-7 cells was done with Rhodamine 123 stain by flow cytometry. (b) The percentage of M1 reflects the reduction of Δψm. All experiments were done independently in triplicate per experimental point, and representative results are shown. All data represent as the mean ±SD of three independent experiments. *p<0.05 and **p<0.01 versus control.

Here, we further evaluated whether THC treatment had any effects on the Δψm, which was critical in cells undergoing apoptosis (23). We monitored the potential gradient across the membrane using the fluorescent dye RH123 (Fig. 5a). As shown in Fig. 5b, when MCF-7 cells were treated with 0, 70, 100 µM THC or 5-FU for 24 h, the population that lost Δψm was increased from 4.1% of the untreated cells to 9.9, 17.4, and 49.6%, respectively (*p*<0.05, *p*<0.01), suggesting that THC could lead to a concentration-dependent dysfunction in mitochondria.

### Effect of THC on caspase-3 and caspase-9 activities in MCF-7 cells

**Fig. 6:**
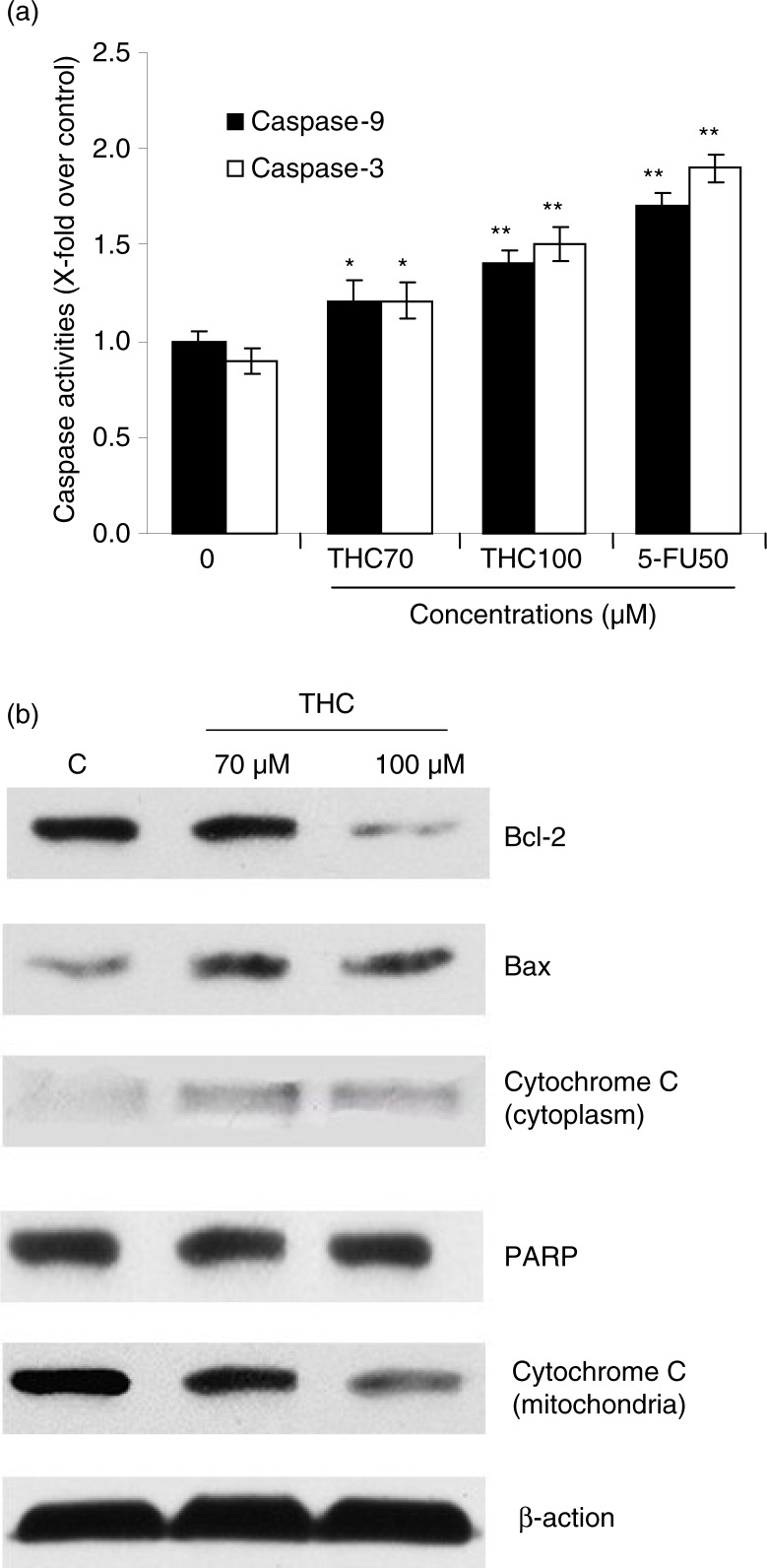
THC-induced mitochondrial apoptotic pathway. (a) Quantitative analysis of caspase-9 activity and caspase-3 activity was performed using synthetic caspase substrates Ac-LEHD-*p*NA and Ac-DEVD-*p*NA. Data in the figure represent mean±SD of three independent experiments. *p<0.05 and **p<0.01 indicate that those groups differ significantly from the control. (b) The protein expression was determined with or without THC by western blot (β-action as loading control). THC-induced upregulation of Bax, cytochrome c, and PARP, and downregulation of Bcl-2 by western blotting assay. MCF-7 cells were treated with THC at 0, 70, and 100 µM for 24 h. The test was repeated three times and representative blots are shown.

Caspase protease family is believed to play a central role in mitochondria-mediated apoptotic responses (18). To investigate the apoptotic pathway in THC-treated MCF-7 cells, we examined the caspase-3 and caspase-9 activities. As showed in Fig. 6a, both caspase-3 and caspase-9 were significantly activated by THC. Meanwhile, the activity of caspase-9 in the MCF-7 cells treated with THC at 70, 100 µM, and 5-FU increased by 1.2-fold (*p*<0.05), 1.4-fold (*p*<0.01), and 1.7-fold (*p*<0.01), respectively, compared to control cells. Analogously, intracellular caspase-3 activity was also enhanced with the treatment of THC at 70 and 100 µM (*p*<0.05). These results demonstrated that the THC-induced activation of caspase-3 or caspase-9 was involved in mitochondria-mediated apoptosis in MCF-7 cells.

### Effects of THC on the expression of apoptosis-related proteins

Mitochondria-mediated apoptosis is the best known intrinsic apoptosis pathway (24). To determine whether the mitochondrial apoptotic proteins was involved in THC-induced apoptosis, expression patterns of pro-apoptotic and anti-apoptotic Bcl-2 family proteins, cytochrome c, and PARP in THC-treated MCF-7 cells were investigated by western blot. As depicted in Fig. 6b, THC at 70 and 100 µM significantly downregulated the expression of anti-apoptotic protein Bcl-2, whereas the expression of pro-apoptotic protein Bax was upregulated with the increasing concentrations of THC treatment. At the same time, the content of cytochrome c in cytosol was also significantly increased, whereas cytochrome c levels in mitochondria was decreased following THC treatment. Moreover, it was also found that THC caused a slight increase in the cleavage of PARP in MCF-7 cells, indicating that mitochondrial pathway is involved in THC-induced apoptosis.

### The role of ROS in THC-induced apoptosis

**Fig. 7:**
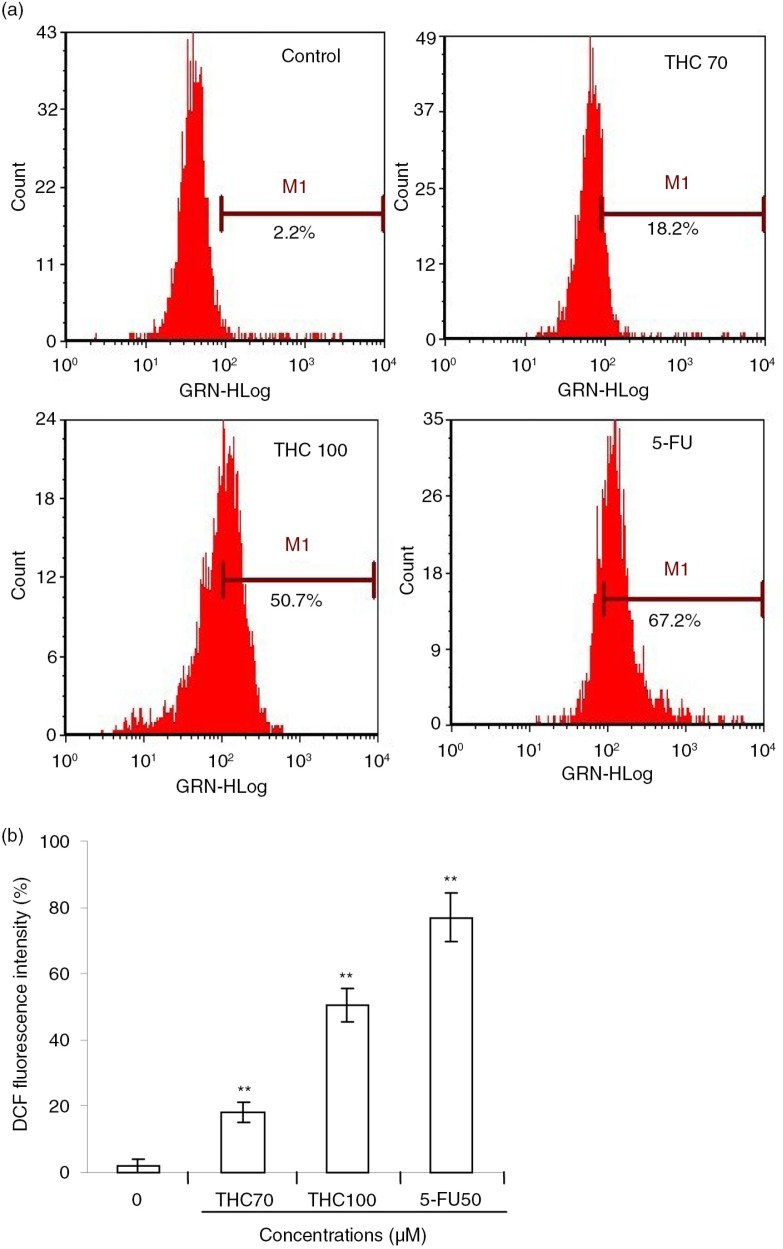
Effects of THC on intracellular ROS generation in MCF-7 cells. (a) MCF-7 cells were treated with 0, 70, 100 µM THC or 5-FU (50 µM) for 24 h, and then stained with DCFH-DA (10 µM), followed by an assay by flow cytometry. (b) ROS in the cells converts the non-fluorescent dye into fluorescein, which emits green fluorescence. Fluorescence intensity is an indication of ROS levels in MCF-7 cells. All data represent the mean ±SD of three independent experiments. **p<0.01 versus control.

The escalated ROS level in the mitochondria can already push cancer cells to the brink of their toxic threshold, which is implicated in the induction of apoptosis by several flavonoids (24). Herein, we speculated that the cell growth inhibition of THC might be partly due to additional increased intracellular ROS generation, perturbing cellular redox homeostasis. To test this hypothesis, we next measured intracellular ROS in terms of fluorescence by DCF. As depicted in Fig. 7a, the green fluorescence intensities of DCF signals were significantly enhanced with the addition of THC. As supposed, 70, 100 µM THC, and 5-FU separately resulted in the production of H_2_O_2_ by 18.2, 50.7, and 67.2%, respectively, in MCF-7 cells, compared with untreated control (2.2%, *p*<0.01, Fig. 7b). The data present here indicated that MCF-7 cells treated with THC exhibited a rapid response in the generation of intracellular H_2_O_2_, relative to basal levels of ROS. These results suggest that THC may induce growth inhibition and apoptosis through enhancing intracellular ROS oxidative stress of MCF-7 cancer cells.

## Discussion

In recent years, it has been reported that curcumin exhibits antineoplastic activities *in vivo* and *in vitro* (25–27). However, the pharmacokinetic features of curcumin in several species have indicated poor systemic bioavailability, which may be related to its inadequate absorption (11). Therefore, we speculate that THC, a key phase I metabolite of curcumin, might be one of the genuine effective forms of curcumin *in vivo* to contribute to the pharmacological effects against human breast cancer. Interestingly, THC has recently been reported to induce autophagy in human leukemia HL-60 cells (28), and it also inhibits HT1080 cell invasion and motility (12). However, there is little knowledge regarding the potency and mechanism of THC against breast cancer. In order to better understand the mechanism of apoptosis induced by THC, we investigated the effects of this compound on human breast cancer MCF-7 cells. Here, the molecular mechanism underlying THC-induced apoptosis is currently being elucidated, and THC was shown to have cytotoxic effect against MCF-7 cells via inducing G0/G1 arrest and mitochondria-mediated apoptosis.

Recent research has demonstrated that flavonoids cause growth inhibition to human cancer cells through interfering with the cell cycle progress (29). It is also well known that apoptosis and cell cycle deregulation are closely related events, and disruption of cell cycle progression may ultimately lead to apoptotic/necrotic death (15). In our cell cycle analysis, THC consistently exhibited effective cell growth inhibition by inducing cancer cells to undergo G0/G1 phase arrest in MCF-7 cells (Fig. 3). However, our findings were not in agreement with other previously reports, which showed that curcumin inhibited the cell cycle progression of gastric cancer cells at G2/M in HL-60 cells and MCF-7 cells cycle (29). Differences in the tested responses are most likely due to the specific cell type and concentration and treatment time used. Furthermore, there is mounting evidence that chemotherapeutic agents induce tumor regression through induction of apoptosis (28). In this study, the apoptosis induction ability of THC was confirmed and quantified by FACS analysis after staining with Annexin V-FITC/PI. A cell population with Annexin V positive and PI negative is considered as an early apoptotic population, whereas a cell population with both annexin V and PI positive is considered as a late apoptotic/necrotic population (30). As depicted in Fig. 4, the results from annexin V/PI co-staining assay clearly demonstrated that THC induced apoptosis in MCF-7 cells, as reflected by the fact that the late apoptotic cells were concomitantly increased with the increased time of THC treatment. This finding was further confirmed by many morphological and biochemical changes that happened during apoptosis, including cell shrinkage, formation of apoptotic bodies, and nuclear condensation in the cancer cells treated with THC (Fig. 4). Our result is in agreement with earlier investigations showing that curcumin causes growth inhibition in human cancer cells by interfering with the cellular apoptosis progress (13).

Mitochondrial pathway is thought to be the major pathway for apoptosis, and therefore, targeting the mitochondria is a novel strategy for cancer therapy (31). Mitochondria-mediated apoptosis is highly regulated by the Bcl-2 family protein comprising both anti-apoptotic (Bcl-2) and pro-apoptotic members (Bax), and the balance between the expression levels of pro-apoptotic and anti-apoptotic proteins is critical for cell survival or cell death (19, 32). Mitochondrial dysfunction usually triggers some cellular signaling to induce apoptosis, and mitochondrial transmembrane potential (Δψm) decreasing and cytochrome c releasing to cytosol are two common parameters of mitochondrial dysfunction (33). As demonstrated by using RH123 staining, there was a decrease in RH123 fluorescence after treatment with THC as early as 24 h in comparison with the untreated cells, indicating that THC-induced Δψm disruption in MCF-7 cells. Meanwhile, we also found that cytochrome c was released from the mitochondria to the cytosol (Fig. 6b). In addition, the caspase protein family is believed to play a central role in mediating various apoptotic responses (34). Caspases include two types of subfamilies, namely upstream initiator caspases (e.g. caspase-8 and capase-9) and downstream effector caspases (e.g. caspase-3), which are responsible for the activation of the executioner caspases within the mitochondrial pathway of apoptosis (34). In the cytoplasm, cytochrome c, together with caspase-9, constitutes a proteolytic active complex called apoptosome to activate caspase-3, and eventually provokes apoptosis (35, 36). It had also been reported that Bax, PARP, caspase-3 and caspase-9 were increased, and Bcl-2 and Δψm were decreased in curcumin-treated MCF-7 cells, leading to apoptosis (37). Here, we found that THC decreased Δψm and Bcl-2 expression (Figs. 5 and 6), and increased Bax expression (Fig. 6b), which could be responsible for THC-induced apoptotic process. Furthermore, cytochrome c was found to be released from mitochondria to the cytosol. It is widely recognized that the cleavage of PARP, which is one of the substrates for caspase-3, is an early and critical event required for tumor cells apoptosis (35). In our hands, the activation of caspase-3 and the cleavage of PARP were clearly observed, indicating that apoptosis induced by THC in MCF-7 cells occurred via mitochondria-dependent signal pathway.

In mammalian cells, increased cellular ROS production has been suggested to be responsible for the depolarization of mitochondrial Δψm and subsequent cell apoptosis, and some flavonoids are recently shown to stimulate cellular oxidative metabolism (38). Mitochondria are well known to be a source of ROS during apoptosis, and previous studies indicate that the ROS generation is an upstream factor for regulating apoptosis (39). To confirm if the mitochondrial dysfunction observed in MCF-7 cells treated with THC was promoted by ROS production, we measured ROS levels using the cell-permeable dye DCF-DA. The results showed that the apoptotic effect of THC against MCF-7 cells was associated with an elevated level of intracellular ROS in a concentration–response manner. As showed in Fig. 7b, the levels of H_2_O_2_ in 70 and 100 µM of THC-treated and 5-Fu-treated cells for 24 h were elevated by 18.2, 50.7, and 76.9%, respectively, compared with the untreated control cells. These results suggest that ROS-dependent mechanism may be involved in THC-induced apoptosis, and ROS-independent mechanism needs to be further investigated in the future.

In conclusion, this is the first study to show that THC, a major metabolite of curcumin, may be one of the active antitumor forms of curcumin, and it is able to exhibit anti-proliferation of human breast cancer MCF-7 cells via cell cycle arrest at the G0/G1 phase and apoptosis induced by ROS-dependent mitochondrial pathway. The observed antitumor effect indicates the possibility that THC may provide an effective and alternative treatment strategy for cancer. Understanding the involvement of the active THC in human cancer treatment is an attractive challenge and requires further investigation.
